# The genome sequence of Blair’s Shoulder-knot, *Lithophane leautieri *(Boisduval, 1829)

**DOI:** 10.12688/wellcomeopenres.22610.1

**Published:** 2024-07-10

**Authors:** David C. Lees, Natural History Museum Genome Acquisition Lab, Darwin Tree of Life Barcoding collective, Wellcome Sanger Institute Tree of Life Management, Samples and Laboratory team, Wellcome Sanger Institute Scientific Operations: Sequencing Operations, Wellcome Sanger Institute Tree of Life Core Informatics team, Tree of Life Core Informatics collective, Darwin Tree of Life Consortium

**Affiliations:** 1 Natural History Museum, London, England, UK

**Keywords:** Lithophane leautieri, Blair’s Shoulder-knot, genome sequence, chromosomal, Lepidoptera

## Abstract

We present a genome assembly from an individual male *Lithophane leautieri* (Blair’s Shoulder-knot; Arthropoda; Insecta; Lepidoptera; Noctuidae). The genome sequence is 521.7 megabases in span. Most of the assembly is scaffolded into 31 chromosomal pseudomolecules, including the Z sex chromosome. The mitochondrial genome has also been assembled and is 15.4 kilobases in length. Gene annotation of this assembly on Ensembl identified 12,254 protein coding genes.

## Species taxonomy

Eukaryota; Opisthokonta; Metazoa; Eumetazoa; Bilateria; Protostomia; Ecdysozoa; Panarthropoda; Arthropoda; Mandibulata; Pancrustacea; Hexapoda; Insecta; Dicondylia; Pterygota; Neoptera; Endopterygota; Amphiesmenoptera; Lepidoptera; Glossata; Neolepidoptera; Heteroneura; Ditrysia; Obtectomera; Noctuoidea; Noctuidae; *Lithophane*; *Lithophane leautieri* (Boisduval, 1829) (NCBI:txid1870035).

## Background

*Lithophane leautieri* (Boisduval, 1829) also known as the Blair’s Shoulder-knot ( Waring *et al.*, 2017), is a moth in family Noctuidae with a wingspan of 40–45 mm and a 17–20 mm forewing length ( Bretherton *et al.*, 1983; Waring *et al.*, 2017). Its narrow forewing is greyish and has a long thin black basal streak; the slanting orbicular and reniform stigmata are obscure and often underscored with a dark streak and sometimes there is a pinkish area around the reniform; the hindwing is fuscous while the thorax is grey and crested ( Bretherton *et al.*, 1983; Waring *et al.*, 2017). *L. leautieri* is confusable with other greyish *Lithophane* species with more distinct forewing stigmata, but it does not roll its wings at rest.

The egg is pearly white, a flattened sphere, and maroon when developed; the green larva (fullfed 36 mm) also has a greenish head and is variegated with a dorsally dashed white stripe and lateral zigzag white stripes which render it particularly well camouflaged amongst the leaves of its cupressaceous foodplants. These are junipers and cypresses. The eggs are laid at leaf tips and the larva eats the buds and blossoms of cypresses such as the introduced Lawson’s cypress *Cupressus lawsoniana*. (A.Murray bis) Parl. and, in gardens, the widely planted hybrid Leyland cypress *Cupressus* x *leylandii* A. B. Jacks. & Dallim. This has greatly contributed to the range expansion in Britain. The olive to chestnut brown pupa is about 17 mm long ( Waring *et al.*, 2017). Blair’s Shoulder-knot is univoltine, flying between mid-September and late November, with a peak in earlier October ( Randle *et al.*, 2019), but unlike many *Lithophane* it does not overwinter as an adult but rather as an egg that hatches in February or March ( Bretherton *et al.*, 1983). The imago is nocturnal and comes readily to light and tends to fly after 23:00 ( Bretherton *et al.*, 1983).

*Lithophane leautieri* was first found in 1951 in the United Kingdom and is now widespread as far north as central Scotland and in eastern Ireland, arriving in the Channel Islands by 1961 ( Randle *et al.*, 2019). As such it has greatly increased in abundance from 1970–2016 by 321% and its distribution from 1980–2016 by 206%, but this expansion seems to have stabilised since 2000 ( Randle *et al.*, 2019). In Europe it is prevalent from southwestern Spain to southern Italy, Greece and Croatia and northeast as far as Denmark ( GBIF Secretariat, 2024; Lepiforum, 2024), but in Europe it is not as widely distributed as it is now within its range in the United Kingdom.

Up to six subspecies have been recognised: *Lithophane leautieri sabinae* (Geyer, [1832]) from Germany (type locality), *L. l. nicaeensis* Boursin, 1957 from S. France, *L. l. cyrnos* Boursin, 1957, *L. l. hesperica* Boursin, 1957 from France (also the one recognised in Britain), *L. l. andalusica* Boursin, 1962 from Spain and *L. l. ochreimacula* (Rothschild, 1914) from Algeria ( Savela, 2024).

The COI-5P portion of the mitochondrion from the genome assembly (OX424489.1) is identical to three DNA barcodes on BOLD (18/04/2024) from France (Normandy, Val de Loire) and three other haplotypes are so far known from the United Kingdom. It is 2.11% distant from a specimen from Italy (TLMF Lep 04600) also representing the BIN cluster BOLD:AAD1371, but it is 2.45–3.23% distant from the mitochondrially diverse BOLD:ABX5694 from Spain and Corsica (the latter would represent ssp. *cyrnos*). Average distance of the BIN BOLD:AAD1371 (18/04/2024) is 0.18%, maximum 0.98% ( *n* = 12) and it is 2.21% distant from BOLD:ABX5694, also identified as *L. leautieri*. *L. leautieri* has been placed in the subgenus *Prolitha* Berio, 1980 along with *L. lapithea* (Hübner, [1808]) from the Mediterranean region ( Lafontaine & Schmidt, 2010). However, the DNA barcode from the mitochondrion assembly is 5.66% distant to *L. pertorrida* (McDunnough, 1942) which has been placed in the *antennata* species-group ( Brou & Lafontaine, 2009) and it is 5.81% distant from *L. antennata* (Walker, 1858) (BOLD:AAB5821, a cluster that also includes specimens identified as *L. grotei* (Riley, 1882), *L. laceyi* (Barnes & McDunnough, 1913) and *L. laticinerea* (Grote, 1874).

The genome will be helpful in further taxonomic and evolutionary work on the genus *Lithophane* Hübner, [1821] including for re-evaluating subspecies concepts in *L. leautieri*.

## Genome sequence report

**Figure 1.**
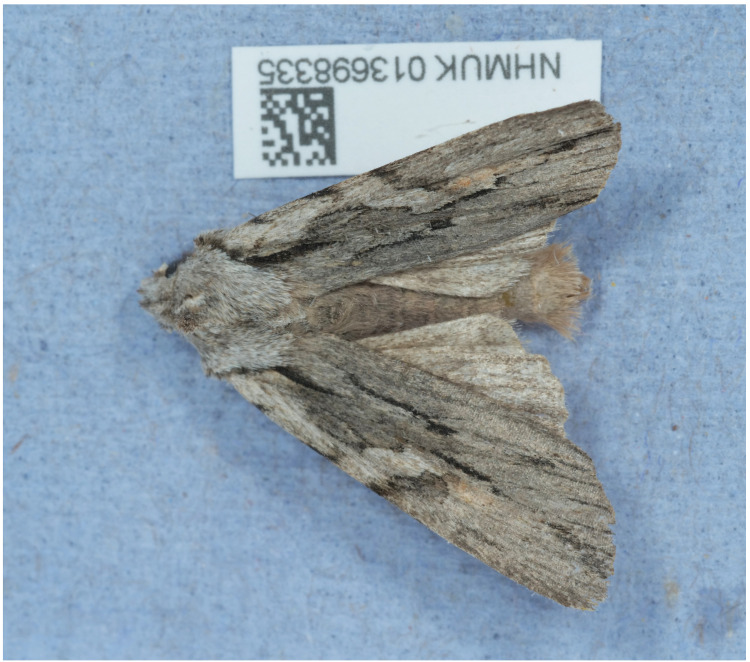
Photograph of the *Lithophane leautieri* (ilLitLeau1) specimen used for genome sequencing.

The genome was sequenced from a male *Lithophane leautieri* ( Figure 1) collected from Lucas Road, High Wycombe, England, UK (51.63, –0.74). A total of 56-fold coverage in Pacific Biosciences single-molecule HiFi long reads was generated. Primary assembly contigs were scaffolded with chromosome conformation Hi-C data. Manual assembly curation corrected 7 missing joins or mis-joins and removed one haplotypic duplication, reducing the scaffold number by 2.17%.

**Figure 2.**
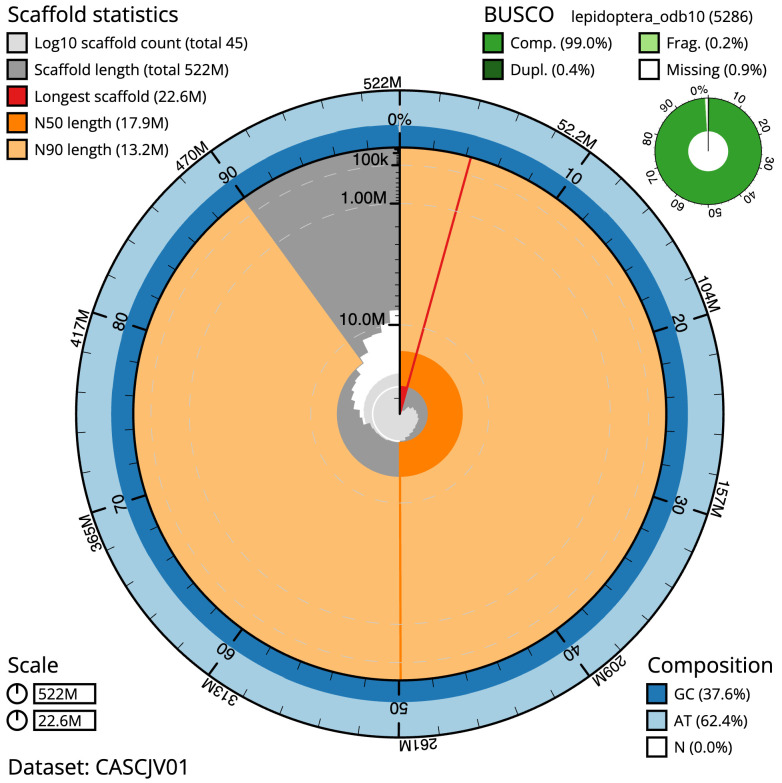
Genome assembly of *Lithophane leautieri*, ilLitLeau1.1: metrics. The BlobToolKit snail plot shows N50 metrics and BUSCO gene completeness. The main plot is divided into 1,000 size-ordered bins around the circumference with each bin representing 0.1% of the 521,693,918 bp assembly. The distribution of scaffold lengths is shown in dark grey with the plot radius scaled to the longest scaffold present in the assembly (22,560,491 bp, shown in red). Orange and pale-orange arcs show the N50 and N90 scaffold lengths (17,894,250 and 13,159,172 bp), respectively. The pale grey spiral shows the cumulative scaffold count on a log scale with white scale lines showing successive orders of magnitude. The blue and pale-blue area around the outside of the plot shows the distribution of GC, AT and N percentages in the same bins as the inner plot. A summary of complete, fragmented, duplicated and missing BUSCO genes in the lepidoptera_odb10 set is shown in the top right. An interactive version of this figure is available at https://blobtoolkit.genomehubs.org/view/CASCJV01/dataset/CASCJV01/snail.

**Figure 3.**
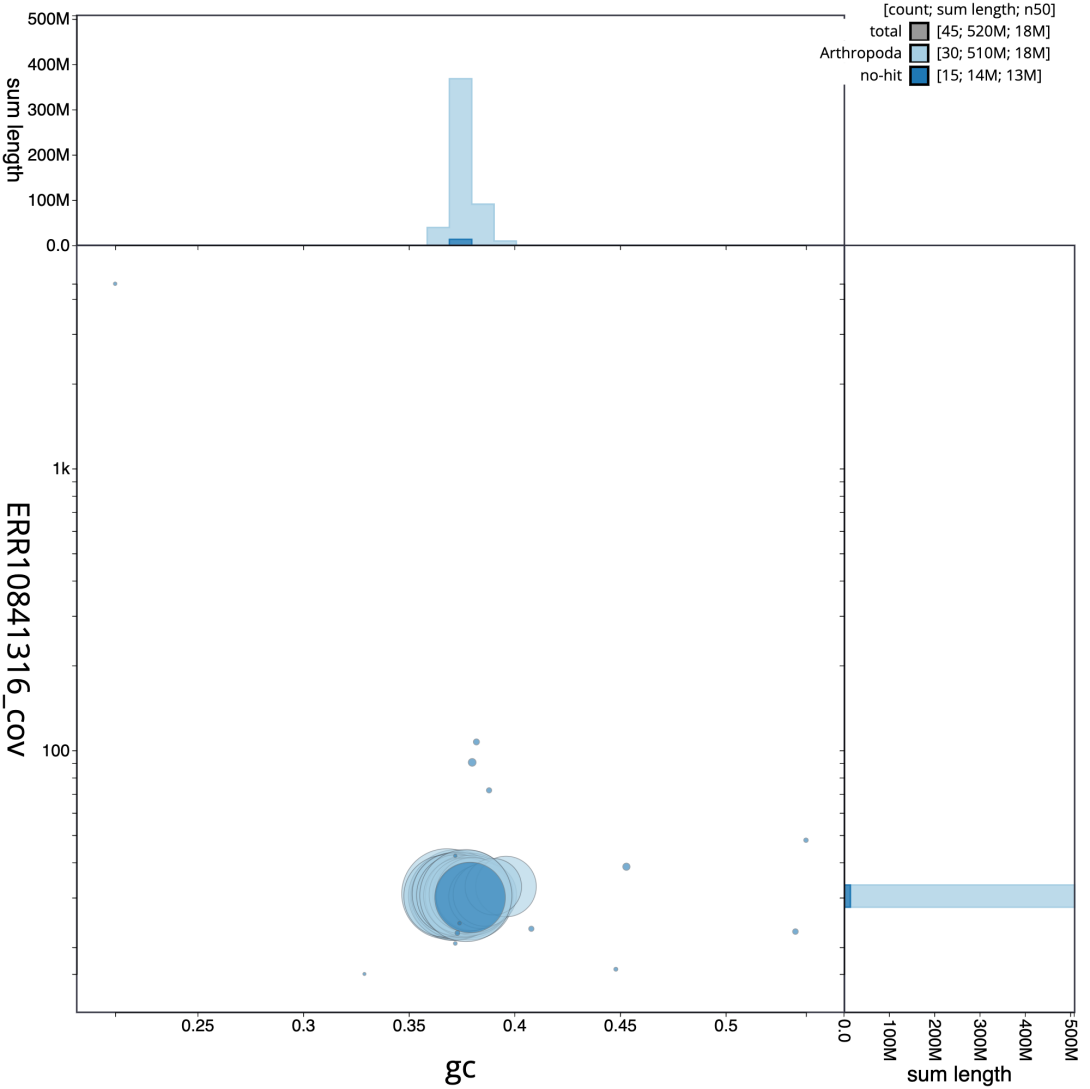
Genome assembly of *Lithophane leautieri*, ilLitLeau1.1: BlobToolKit GC-coverage plot. Scaffolds are coloured by phylum. Circles are sized in proportion to scaffold length. Histograms show the distribution of scaffold length sum along each axis. An interactive version of this figure is available at https://blobtoolkit.genomehubs.org/view/CASCJV01/dataset/CASCJV01/blob.

**Figure 4.**
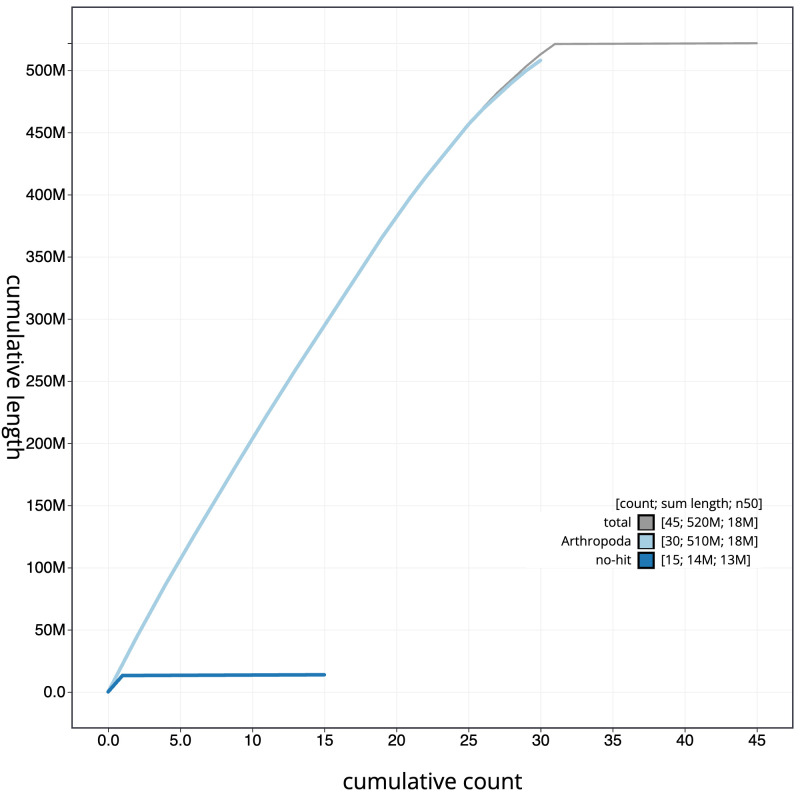
Genome assembly of *Lithophane leautieri*, ilLitLeau1.1: BlobToolKit cumulative sequence plot. The grey line shows cumulative length for all scaffolds. Coloured lines show cumulative lengths of scaffolds assigned to each phylum using the buscogenes taxrule. An interactive version of this figure is available at https://blobtoolkit.genomehubs.org/view/CASCJV01/dataset/CASCJV01/cumulative.

**Figure 5.**
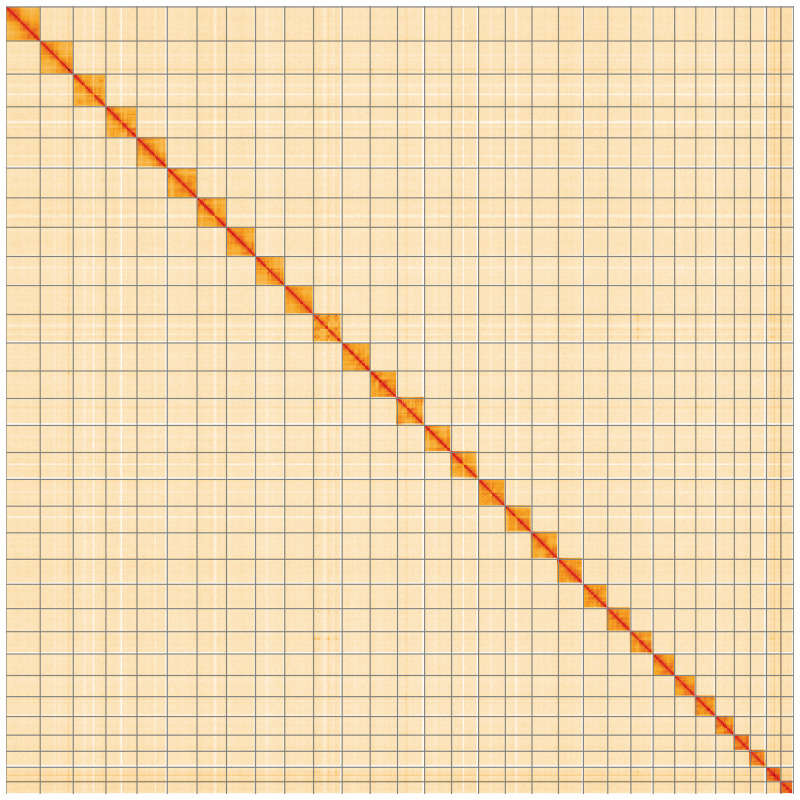
Genome assembly of *Lithophane leautieri*, ilLitLeau1.1: Hi-C contact map of the ilLitLeau1.1 assembly, visualised using HiGlass. Chromosomes are shown in order of size from left to right and top to bottom. An interactive version of this figure may be viewed at https://genome-note-higlass.tol.sanger.ac.uk/l/?d=YkBcWlMPSG-pwbANlgoFfw.

The final assembly has a total length of 521.7 Mb in 44 sequence scaffolds with a scaffold N50 of 17.9 Mb ( Table 1). The snail plot in Figure 2 provides a summary of the assembly statistics, while the distribution of assembly scaffolds on GC proportion and coverage is shown in Figure 3. The cumulative assembly plot in Figure 4 shows curves for subsets of scaffolds assigned to different phyla. Most (99.88%) of the assembly sequence was assigned to 31 chromosomal-level scaffolds, representing 30 autosomes and the Z sex chromosome. Chromosome-scale scaffolds confirmed by the Hi-C data are named in order of size ( Figure 5; Table 2). The Z chromosome was identified based on synteny with *Lithophane semibrunnea* (GCA_947363905.1). While not fully phased, the assembly deposited is of one haplotype. Contigs corresponding to the second haplotype have also been deposited. The mitochondrial genome was also assembled and can be found as a contig within the multifasta file of the genome submission.

**Table 1.** Genome data for *Lithophane leautieri*, ilLitLeau1.1.

Project accession data		
Assembly identifier	ilLitLeau1.1	
Species	*Lithophane leautieri*	
Specimen	ilLitLeau1	
NCBI taxonomy ID	1870035	
BioProject	PRJEB59370	
BioSample ID	Genome and RNA sequencing: SAMEA111458113 Hi-C scaffolding: SAMEA111458102	
Isolate information	ilLitLeau1: thorax (genome sequencing and RNA) ilLitLeau1: head (Hi-C sequencing)	
Assembly metrics *		*Benchmark*
Consensus quality (QV)	69.8	≥ *50*
*k*-mer completeness	100.0%	≥ *95%*
BUSCO **	C:99.0%[S:98.5%,D:0.4%],F:0.2%,M:0.9%,n:5,286	*C* ≥ *95%*
Percentage of assembly mapped to chromosomes	99.88%	≥ *95%*
Sex chromosomes	Z	*localised homologous pairs*
Organelles	Mitochondrial genome: 15.4 kb	*complete single alleles*
Raw data accessions		
PacificBiosciences SEQUEL II	ERR10841316	
Hi-C Illumina	ERR10851509	
PolyA RNA-Seq Illumina	ERR11242523	
Genome assembly		
Assembly accession	GCA_949152455.1	
*Accession of alternate haplotype*	GCA_949152515.1	
Span (Mb)	521.7	
Number of contigs	94	
Contig N50 length (Mb)	9.7	
Number of scaffolds	44	
Scaffold N50 length (Mb)	17.9	
Longest scaffold (Mb)	22.56	
Genome annotation		
Number of protein-coding genes	12,254	
Number of non-coding genes	1,703	
Number of gene transcripts	22,160	

* Assembly metric benchmarks are adapted from column VGP-2020 of “Table 1: Proposed standards and metrics for defining genome assembly quality” from ( Rhie *et al.*, 2021). ** BUSCO scores based on the lepidoptera_odb10 BUSCO set using version 5.3.2. C = complete [S = single copy, D = duplicated], F = fragmented, M = missing, n = number of orthologues in comparison. A full set of BUSCO scores is available at https://blobtoolkit.genomehubs.org/view/CASCJV01/dataset/CASCJV01/busco.

**Table 2.** Chromosomal pseudomolecules in the genome assembly of *Lithophane leautieri*, ilLitLeau1.

INSDC accession	Chromosome	Length (Mb)	GC%
OX424459.1	1	21.88	37.0
OX424460.1	2	21.63	37.0
OX424461.1	3	20.56	38.0
OX424462.1	4	20.0	38.0
OX424463.1	5	19.72	37.0
OX424464.1	6	19.49	37.0
OX424465.1	7	19.28	37.5
OX424466.1	8	19.26	37.0
OX424467.1	9	19.1	37.5
OX424468.1	10	18.86	37.5
OX424469.1	11	18.56	37.0
OX424470.1	12	18.15	37.5
OX424471.1	13	17.89	37.5
OX424472.1	14	17.89	37.5
OX424473.1	15	17.77	36.5
OX424474.1	16	17.75	37.0
OX424475.1	17	17.65	37.5
OX424476.1	18	17.49	37.5
OX424477.1	19	16.52	38.0
OX424478.1	20	16.17	38.0
OX424479.1	21	15.27	37.5
OX424480.1	22	14.7	38.0
OX424481.1	23	14.26	37.5
OX424482.1	24	14.02	38.0
OX424483.1	25	13.16	38.0
OX424484.1	26	12.28	38.0
OX424485.1	27	10.67	38.5
OX424486.1	28	10.57	38.5
OX424487.1	29	9.65	39.5
OX424488.1	30	8.34	39.0
OX424458.1	Z	22.56	37.5
OX424489.1	MT	0.02	21.5

The estimated Quality Value (QV) of the final assembly is 69.8 with *k*-mer completeness of 100.0%, and the assembly has a BUSCO v5.3.2 completeness of 99.0% (single = 98.5%, duplicated = 0.4%), using the lepidoptera_odb10 reference set ( *n* = 5,286).

Metadata for specimens, barcode results, spectra estimates, sequencing runs, contaminants and pre-curation assembly statistics are given at https://links.tol.sanger.ac.uk/species/1870035.

## Genome annotation report

The *Lithophane leautieri* genome assembly (GCA_949152455.1) was annotated at the European Bioinformatics Institute (EBI) on Ensembl Rapid Release. The resulting annotation includes 22,160 transcribed mRNAs from 12,254 protein-coding and 1,703 non-coding genes ( Table 1; https://rapid.ensembl.org/Lithophane_leautieri_GCA_949152455.1/Info/Index).

## Methods

### Sample acquisition and nucleic acid extraction

A male *Lithophane leautieri* (specimen ID NHMUK013698335, ToLID ilLitLeau1) was collected from Lucas Road, High Wycombe, England, UK (latitude 51.63, longitude –0.74) on 2021-10-27. The specimen was collected and identified by David Lees (Natural History Museum) and preserved by dry freezing at –80° C.

Protocols developed by the Wellcome Sanger Institute (WSI) Tree of Life core laboratory have been deposited on protocols.io ( Denton *et al.*, 2023b). The workflow for high molecular weight (HMW) DNA extraction at the WSI includes a sequence of core procedures: sample preparation; sample homogenisation, DNA extraction, fragmentation, and clean-up. In sample preparation, the ilLitLeau1 sample was weighed and dissected on dry ice ( Jay *et al.*, 2023). Dry Pulverizer ( Narváez-Gómez *et al.*, 2023). Tissue from the thorax was homogenised using a PowerMasher II tissue disruptor ( Denton *et al.*, 2023a). HMW DNA was extracted using the Automated MagAttract v1 protocol ( Oatley *et al.*, 2023). HMW DNA was sheared into an average fragment size of 12–20 kb in a Megaruptor 3 system with speed setting 30 ( Todorovic *et al.*, 2023). Sheared DNA was purified by solid-phase reversible immobilisation ( Strickland *et al.*, 2023): in brief, the method employs a 1.8X ratio of AMPure PB beads to sample to eliminate shorter fragments and concentrate the DNA. The concentration of the sheared and purified DNA was assessed using a Nanodrop spectrophotometer and Qubit Fluorometer and Qubit dsDNA High Sensitivity Assay kit. Fragment size distribution was evaluated by running the sample on the FemtoPulse system.

RNA was extracted from thorax tissue of ilLitLeau1 in the Tree of Life Laboratory at the WSI using the RNA Extraction: Automated MagMax™ *mir*Vana protocol ( do Amaral *et al.*, 2023). The RNA concentration was assessed using a Nanodrop spectrophotometer and a Qubit Fluorometer using the Qubit RNA Broad-Range Assay kit. Analysis of the integrity of the RNA was done using the Agilent RNA 6000 Pico Kit and Eukaryotic Total RNA assay.

### Sequencing

Libraries were prepared and sequencing performed by the Scientific Operations core at the WSI. Pacific Biosciences HiFi circular consensus DNA sequencing libraries were constructed according to the manufacturers’ instructions. Poly(A) RNA-Seq libraries were constructed using the NEB Ultra II RNA Library Prep kit. DNA and RNA sequencing was performed by the Scientific Operations core at the WSI on Pacific Biosciences SEQUEL II (HiFi) and Illumina NovaSeq 6000 (RNA-Seq) instruments. Hi-C data were also generated from head tissue of ilLitLeau1 using the Arima v2 kit. The Hi-C sequencing was performed using paired-end sequencing with a read length of 150 bp on the Illumina NovaSeq 6000 instrument.

### Genome assembly, curation and evaluation

Assembly was carried out with Hifiasm ( Cheng *et al.*, 2021) and haplotypic duplication was identified and removed with purge_dups ( Guan *et al.*, 2020). The assembly was then scaffolded with Hi-C data ( Rao *et al.*, 2014) using YaHS ( Zhou *et al.*, 2023). The assembly was checked for contamination and corrected as described previously ( Howe *et al.*, 2021). Manual curation was performed using HiGlass ( Kerpedjiev *et al.*, 2018) and Pretext ( Harry, 2022). The mitochondrial genome was assembled using MitoHiFi ( Uliano-Silva *et al.*, 2023), which runs MitoFinder ( Allio *et al.*, 2020) or MITOS ( Bernt *et al.*, 2013) and uses these annotations to select the final mitochondrial contig and to ensure the general quality of the sequence.

A Hi-C map for the final assembly was produced using bwa-mem2 ( Vasimuddin *et al.*, 2019) in the Cooler file format ( Abdennur & Mirny, 2020). To assess the assembly metrics, the *k*-mer completeness and QV consensus quality values were calculated in Merqury ( Rhie *et al.*, 2020). This work was done using Nextflow ( Di Tommaso *et al.*, 2017) DSL2 pipelines “sanger-tol/readmapping” ( Surana *et al.*, 2023a) and “sanger-tol/genomenote” ( Surana *et al.*, 2023b). The genome was analysed within the BlobToolKit environment ( Challis *et al.*, 2020) and BUSCO scores ( Manni *et al.*, 2021; Simão *et al.*, 2015) were calculated.

Table 3 contains a list of relevant software tool versions and sources.

**Table 3.** Software tools: versions and sources.

Software tool	Version	Source
BlobToolKit	4.1.7	https://github.com/blobtoolkit/blobtoolkit
BUSCO	5.3.2	https://gitlab.com/ezlab/busco
Hifiasm	0.16.1-r375	https://github.com/chhylp123/hifiasm
HiGlass	1.11.6	https://github.com/higlass/higlass
Merqury	MerquryFK	https://github.com/thegenemyers/MERQURY.FK
MitoHiFi	2	https://github.com/marcelauliano/MitoHiFi
PretextView	0.2	https://github.com/sanger-tol/PretextView
purge_dups	1.2.3	https://github.com/dfguan/purge_dups
sanger-tol/genomenote	v1.0	https://github.com/sanger-tol/genomenote
sanger-tol/readmapping	1.1.0	https://github.com/sanger-tol/readmapping/tree/1.1.0
YaHS	1.2a	https://github.com/c-zhou/yahs

### Genome annotation

The Ensembl Genebuild annotation system ( Aken *et al.*, 2016) was used to generate annotation for the *Lithophane leautieri* assembly (GCA_949152455.1) in Ensembl Rapid Release at the EBI. Annotation was created primarily through alignment of transcriptomic data to the genome, with gap filling via protein-to-genome alignments of a select set of proteins from UniProt ( UniProt Consortium, 2019).

### Wellcome Sanger Institute – Legal and Governance

The materials that have contributed to this genome note have been supplied by a Darwin Tree of Life Partner. The submission of materials by a Darwin Tree of Life Partner is subject to the **‘Darwin Tree of Life Project Sampling Code of Practice’**, which can be found in full on the Darwin Tree of Life website here. By agreeing with and signing up to the Sampling Code of Practice, the Darwin Tree of Life Partner agrees they will meet the legal and ethical requirements and standards set out within this document in respect of all samples acquired for, and supplied to, the Darwin Tree of Life Project.

Further, the Wellcome Sanger Institute employs a process whereby due diligence is carried out proportionate to the nature of the materials themselves, and the circumstances under which they have been/are to be collected and provided for use. The purpose of this is to address and mitigate any potential legal and/or ethical implications of receipt and use of the materials as part of the research project, and to ensure that in doing so we align with best practice wherever possible. The overarching areas of consideration are:

•      Ethical review of provenance and sourcing of the material

•      Legality of collection, transfer and use (national and international)

Each transfer of samples is further undertaken according to a Research Collaboration Agreement or Material Transfer Agreement entered into by the Darwin Tree of Life Partner, Genome Research Limited (operating as the Wellcome Sanger Institute), and in some circumstances other Darwin Tree of Life collaborators.

## Data Availability

European Nucleotide Archive: *Lithophane leautieri*. Accession number PRJEB59370; https://identifiers.org/ena.embl/PRJEB59370 ( Wellcome Sanger Institute, 2023). The genome sequence is released openly for reuse. The *Lithophane leautieri* genome sequencing initiative is part of the Darwin Tree of Life (DToL) project. All raw sequence data and the assembly have been deposited in INSDC databases. Raw data and assembly accession identifiers are reported in Table 1.

## Author information

Members of the Natural History Museum Genome Acquisition Lab are listed here: https://doi.org/10.5281/zenodo.7139035.

Members of the Darwin Tree of Life Barcoding collective are listed here: https://doi.org/10.5281/zenodo.4893703.

Members of the Wellcome Sanger Institute Tree of Life Management, Samples and Laboratory team are listed here: https://doi.org/10.5281/zenodo.10066175.

Members of Wellcome Sanger Institute Scientific Operations: Sequencing Operations are listed here: https://doi.org/10.5281/zenodo.10043364.

Members of the Wellcome Sanger Institute Tree of Life Core Informatics team are listed here: https://doi.org/10.5281/zenodo.10066637.

Members of the Tree of Life Core Informatics collective are listed here: https://doi.org/10.5281/zenodo.5013541.

Members of the Darwin Tree of Life Consortium are listed here: https://doi.org/10.5281/zenodo.4783558.
