# Supplementary material for: The genome sequence of Blair’s Shoulder-knot, Lithophane leautieri (Boisduval, 1829)
Source: Wellcome Open Res. 2024 Jul 10;9:369. [Version 1] doi: 10.12688/wellcomeopenres.22610.1 (PMC11459115; doi:10.12688/wellcomeopenres.22610.1)
Supplement: Peer review history

# Reviewer report: Reviewer response for version 1

**DOI:** 10.21956/wellcomeopenres.24909.r96219

**Published:** 2024-10-07

**Authors:** Joaquin Baixeras

**Affiliations:** 1 Universitat de Valencia, Paterna, Valencia, Spain

This is one of the first reports of the genome assembly of a species of European Lepidoptera. It is a descriptive paper on the result of assembly and general annotation. The research is well organized and looks a relatively clear and well-crafted manuscript. If the editor is thinking in reducing the length, the "background" area may be reduced as this is a common moth, familiar to any interested in Lepidoptera. The results of the genome assembly are shown through the BlobToolKit tools and there is information to interpret the results (see in the references) including interactive access to the graphic material. However, given the fast evolution of genomic tools there is no guarantee that this interaction will be maintained in the future. So, I suggest providing more detailed and self-explanatory information in the legends of the figures (specially Figure3). The reader should not be expected to be familiar with blobtoolkit.

Are sufficient details of methods and materials provided to allow replication by others?

Yes

Is the rationale for creating the dataset(s) clearly described?

Yes

Are the datasets clearly presented in a useable and accessible format?

Yes

Are the protocols appropriate and is the work technically sound?

Yes

Reviewer Expertise:

Entomology, Lepidoptera

I confirm that I have read this submission and believe that I have an appropriate level of expertise to confirm that it is of an acceptable scientific standard.

# Reviewer report: Reviewer response for version 1

**DOI:** 10.21956/wellcomeopenres.24909.r92945

**Published:** 2024-09-04

**Authors:** Hui Wang

**Affiliations:** 1 School of Science, Anhui University of Science and Technology, Huainan, China

Lepidopteran species generally have large and highly repetitive genomes, which poses challenges to the sequencing and assembly of their genomes. This article describes the experimental and analytical processes in a clear and concise manner, and employs innovative methods and strategies that significantly enhance the reliability of the data. It is recommended to accept and indexing this article.

Are sufficient details of methods and materials provided to allow replication by others?

Yes

Is the rationale for creating the dataset(s) clearly described?

Yes

Are the datasets clearly presented in a useable and accessible format?

Yes

Are the protocols appropriate and is the work technically sound?

Yes

Reviewer Expertise:

分子生态学

I confirm that I have read this submission and believe that I have an appropriate level of expertise to confirm that it is of an acceptable scientific standard.
